# Comparison of Efficacy of Topical Carica papaya Leaf Extract and Hemocoagulase in Postoperative Wound Healing After Therapeutic Orthodontic Premolar Extractions: a Split Mouth Study

**DOI:** 10.7759/cureus.61946

**Published:** 2024-06-08

**Authors:** Goutham Vijayakumar, Gidean A Sundaram, Santhosh P Kumar, Saravanan Lakshmanan, Murugesan Krishnan, Vinod K Krishna

**Affiliations:** 1 Oral and Maxillofacial Surgery, Saveetha Dental College and Hospitals, Saveetha Institute of Medical and Technical Sciences, Saveetha University, Chennai, IND

**Keywords:** remodeling, orthodontic therapeutic extractions, innovative technique, dental extraction, wound healing, hemocoagulase, carica papaya leaf extract

## Abstract

Introduction

Postoperative wound healing is the most important factor in the outcome of any surgical procedure. Wound healing is a dynamic process involving inflammation, neovascularization, granulation, fibroblast proliferation, re-epithelization, and remodeling. It repairs tissue integrity, restoring the body's natural defense barrier. A hastened wound healing will help in the quicker re-establishment of the body’s homeostasis. *Carica papaya* includes vital nutrients and bioactive substances such as minerals, vitamins, and antioxidants. Its primary active ingredient papain causes the enzymatic debridement of wounds. Hemocoagulase is a thrombin-like serine protease that is mostly employed for its procoagulant and wound-healing characteristics. It is derived from the venom of *Bothrops *species of snakes. This study aims to compare the wound-healing properties of topical *Carica papaya* leaf extract and Hemocoagulase after dental extractions.

Materials & Methods

For 48 patients requiring bilateral therapeutic dental extraction for orthodontic intervention, *Carica papaya* leaf extract (Caripill 275mg/5ml) was topically applied to the extraction socket on one side, and Hemocoagulase 0.2 CU solution (Botroclot) was applied to the extraction socket on the other side. The bilateral premolars were extracted for orthodontic treatment under local anesthesia. Patients were asked to apply the solution topically twice daily for seven days and were called for review on the seventh day. The assessment of the efficacy of both solutions in post-operative wound healing was the objective of the study. Healing was assessed by using a blinded single observer for all patients using Landry’s healing index.

Results

A total of 48 subjects with 96 sites completed the study, with a mean age of 15.4 years. The study population consisted of 24 males and 24 females, which were evenly distributed among the two study groups. On comparison of wound healing index (WHI) scores between the two groups using the Wilcoxon signed rank test, Group A had a significantly higher mean rank than Group B with regards to the wound healing index score, and the results were statistically significant (p = 0.037).

Conclusion

It can be concluded from the study that *Carica papaya* leaf extract showed better wound healing in post-extraction sockets compared to Hemocoagulase. This study presents the promising use of natural extracts such as *Carica papaya* in wound healing because they are easily accessible to patients, more economical, and have no adverse reactions. More studies that focus on natural extracts to promote wound healing are required in the future.

## Introduction

After a wound, the epithelium loses its continuity, making it vulnerable to various infectious microorganisms. Acute wounds undergo four phases: hemostasis, inflammation, proliferation, and remodeling. Any dysregulation in these steps can delay wound healing. Wound healing can occur with either a primary or secondary intention. Primary intention healing often occurs within 12-24 hours and is commonly observed in wounds where the surgical procedure has successfully approximated the wound margins close together. Healing by secondary intention occurs in wounds that have experienced a substantial loss of soft tissue. The process of epithelial cell regeneration alone is insufficient to completely repair the deficiency. As a result, there is a significant growth of granulation tissue coming from the wound's margin, along with fibrosis. In primary healing, the process of epithelial regeneration is more dominant than fibrosis [1].

In dental treatment, tooth extractions are a commonly performed procedure that most of the population undergoes at least once in their lifetime. Extraction sockets in the oral cavity heal by secondary intention. In the oral cavity, there can be dysregulation of these steps because the oral cavity is constantly exposed to a higher bacterial load than any part of the body. Also, a wound in the oral cavity has to endure nociceptive stimuli due to the temperature of the food being consumed and constant mechanical stimuli due to mastication and phonation. All these factors make a wound in the oral cavity much more challenging for the body to heal [2].

*Carica papaya* includes vital nutrients and bioactive substances such as minerals, vitamins, and antioxidants. Its primary active ingredient, papain, causes the enzymatic debridement of wounds. Vitamin C is necessary for the transformation of proline into hydroxyproline in the granulation tissue of the wounds, which helps to improve wound healing. However, its effect on topical application after dental extractions has not been studied [3]. 

Hemocoagulase is a thrombin-like serine protease that is mostly employed for its procoagulant and wound-healing characteristics. It is derived from the venom of Bothrops species of snakes. At the location of the hemorrhage, it aids in converting prothrombin to thrombin by activating factor Xa. It also stabilizes the fibrin by interacting with factor XIIIa [4]. Both of these agents are natural extracts known to improve wound healing individually; however, their wound-healing abilities have not been compared following dental extractions in humans.

Hence, this study aimed to compare the effects of *Carica papaya* leaf extract and Hemocoagulase in the wound healing process after orthodontic extractions.

## Materials and methods

Study design and setting:

This prospective comparative study was conducted with 48 patients on 96 sites in the Department of Oral and Maxillofacial Surgery, Saveetha Dental College and Hospital in Chennai from March 2023 to October 2023. Following a thorough history-taking, patients underwent a clinical examination and were informed about the procedure, potential complications, and the study's follow-up period. This was approved by the institutional Human Ethical Committee (IHEC/SDC/OMFS-2205/23/257).

Inclusion criteria

Patients requiring bilateral therapeutic dental extraction for orthodontic intervention were included in the study. Maxillary 1st premolars, which required therapeutic extractions, were included in the study.

Exclusion criteria

Dental caries in the teeth to be extracted, gingivitis or periodontitis, hypersensitivity to Hemocoagulase, and third molars requiring therapeutic extractions were excluded.

Surgical protocol

**Figure 1:**
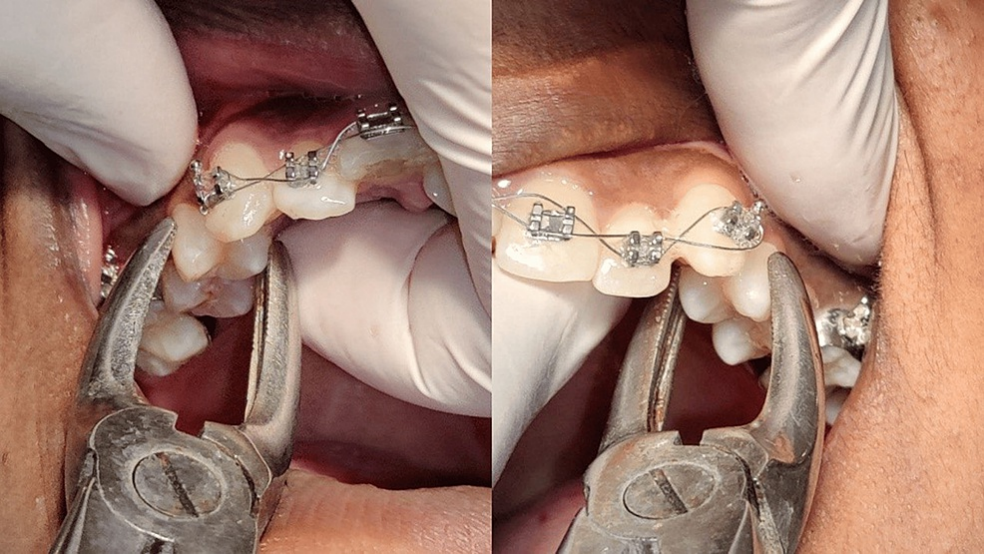
Bilateral therapeutic extractions Right maxillary 1st premolar extraction (right), left maxillary first premolar extraction (left)

Forceps extraction was done under local anesthesia (2% lignocaine with 1:200000 adrenaline). Figure 1 depicts forceps extraction of bilateral maxillary 1st premolars for orthodontic treatment. Group 1 was given 2-3 ml of *Carica papaya* leaf extract (Caripill 275mg/5ml), and Group 2 was given 0.2 CU Hemocoagulase solution (Botroclot). Treatments were topically applied in a gauze piece (2 ml solution/gauze) and the patients were instructed to keep the gauze in place bilaterally for 30 minutes.

**Figure 2:**
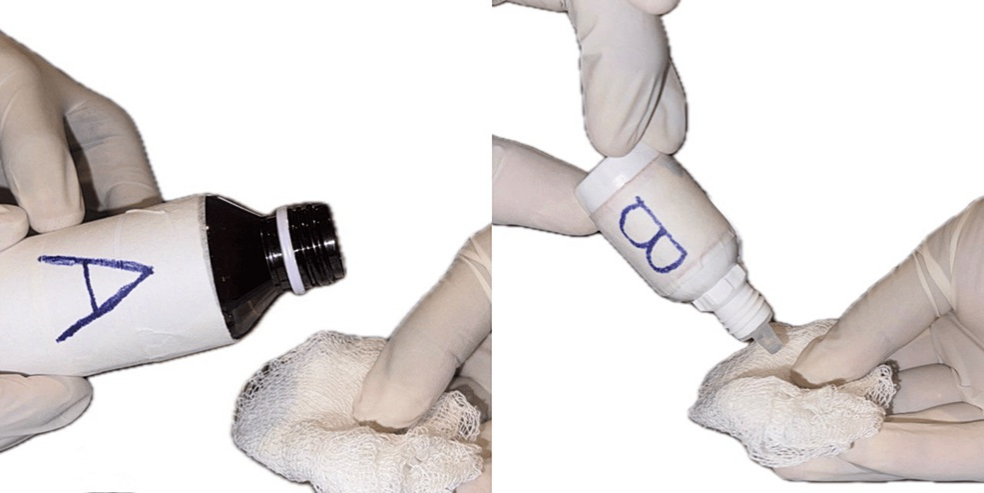
Application of solution on the gauze *Carica papaya* leaf extract labeled -A, Hemocoagulase labeled -B

Figure 2 depicts the application of the solutions from labeled containers on gauze. 

As a standard, left-sided extraction sockets were used for Group 1, and right-sided extraction sockets were used for Group 2. Standard postoperative instructions were given to all patients. Patients were given labeled solution containers on the left and right sides for their reference. Patients were also reminded by phone call to apply their respective solutions topically twice daily for 7 days and were recalled for review on the 8th day. Healing was assessed by using a blinded single observer for all patients using Landry’s healing index (Table 1).

**Table 1:** Wound healing index (Landry et al., 1988) [5]

Healing index	Tissue color	Bleeding on palpation	Granulation tissue	Incision margin	Suppuration
1 - Very poor two or more signs are present	≥ 50% of red gingiva	Yes	Yes	Not epithelized, with loss of epithelium beyond the incision margin	Yes
2 - Poor	≥ 50% of red gingiva	Yes	Yes	Not epithelized, with exposed connective tissue	No
3 - Good	25–50% of red gingiva	No	No	No exposed connective tissue	No
4 - Very good	<25% of red gingiva	No	No	No exposed connective tissue	No
5 - Excellent	All pink tissues	No	No	No exposed connective tissue	No

Statistical analysis

In both groups, the clinical and histological scoring system was represented by numbers and percentages. A p-value of less than 0.05 was deemed significant when using the Wilcoxon signed rank test as a significance test. Additionally, the z-score was determined.

## Results

**Figure 3:**
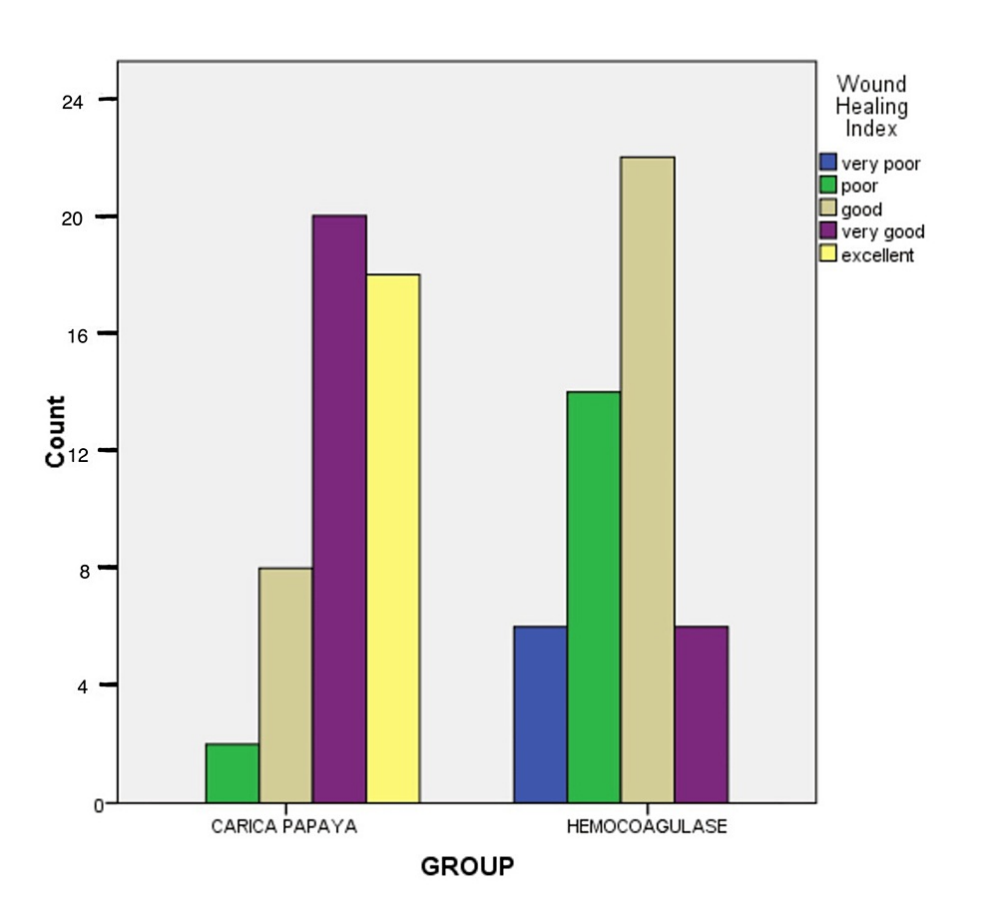
Comparison of wound healing index scores between the two groups

A total of 48 subjects with 96 sites completed the study, with a mean age of 15.4 years. The study population consisted of 24 males and 24 females, which were evenly distributed among the two study groups. On comparison of wound healing index (WHI) scores between the two groups using the Wilcoxon signed rank test, Group A had a significantly higher mean rank than Group B with regards to the wound healing index score, and the results were statistically significant (p = 0.037) (Table 2). In the *Carica papaya *group, a total of 18 subjects had a score of 5 (excellent healing), and 20 subjects had a score of 4 (very good healing). Whereas in the Hemocoagulase group, no subject scored 5 (excellent healing), and only 6 subjects had a score of 4 (very good healing). Conversely, in the *Carica papaya* group, there were no subjects with a score of 1 (very poor healing), and the Hemocoagulase group had 6 subjects with a score of 1 (very poor healing) (Figure 3).

**Table 2:** Comparison of wound healing index scores between the two groups Wilcoxon signed rank test; * - statistically significant

Groups		N	Mean Rank	Z	p-value
Wound Healing Index	Carica Papaya	44	33.73	-4.714	0.037^*^
	Hemocoagulase	44	15.27		

## Discussion

Postoperative wound healing is the most important factor in the outcome of any surgical procedure. Wound healing is a dynamic process involving inflammation, neovascularization, granulation, fibroblast proliferation, re-epithelization, and remodeling. It repairs tissue integrity, restoring the body's natural defense barrier. A hastened wound healing will help in the quicker reestablishment of the body’s homeostasis. In dental extractions, this is of utmost importance because it can restore the function of the patient a lot faster [6].

During wound healing, the void left by the devitalized tissue is replaced by the body, and the wound is restored. Wounds may be healed by primary means if the wound margins are approximated by sutures, or by secondary means if the wound is left to granulate on its own. It depends on whether the wound is sutured or permitted to heal naturally [7,8]. Regrowth of the epithelium is demonstrated where connective tissue development repairs the wounded tissue. Fibroblasts are an active part of wound healing because they both break down the fibrin clot created during injury and replace it with a newly laid extracellular matrix [9,10].

Drugs that reduce pain, control bleeding, and stimulate epithelization have all been widely utilized to speed up the healing process. Numerous studies have looked into how well natural extracts may treat wounds. Such substances include Hemocoagulase and papaya leaf extract [11,12]. Hemocoagulase increases the quality of the clot by activating factors Xa and XIIIa. It increases re-epithelization and collagen production, which speeds up the healing process in extraction sockets [13].

Madhu CP et al. published a study in 2019 that compared healing rates between patients who received topical Hemocoagulase therapy and those who received standard wound dressing. They did this by comparing the size of the wounds in the two groups and found that the study group had better healing rates because the epithelium formed faster [14].

A lot of research on assessing the wound healing benefits of natural substances such as *Curcuma longa, Fumaria vaillantii loisel, Vitis vinifera, Aloe vera, *and* Euterpe oleracea* has been published. Parts of the *Carica papaya* plant, such as the leaf, seed, fruit, and stem, have been used as traditional remedies for many bodily ailments. *Carica papaya* leaf extract has been used in the treatment of dengue fever, where it has been shown to have inhibitory activity against the NS2B-NS3 serine protease, which is necessary for viral replication [15].

A study by Nayak BS et al. compared applying papaya seed extract topically to a wound in Sprague-Dawley rats versus Mupirocin and a control group. The wounds were circular, full-thickness, and left open. The researchers then monitored the wound healing by measuring surface area on alternative days for 13 days, and finally, a histological study and hydroxyproline estimation were made. In this study, rats that were treated with topical *C. papaya* exhibited a notable enhancement in wound-healing activity compared to the other groups. There was more wound contraction, higher levels of hydroxyproline, a lot of collagen deposition, and more fibroblast activity, all of which were signs of this improvement [16].

*Carica papay*a leaf has antioxidant properties such as chelating iron and scavenging hydroxyl. These contribute to their enhanced wound healing by reducing oxidative damage to the cells and preventing destruction by inflammatory mediators in the body.* Carica papaya *extract has been shown to have antibacterial activity against both gram-positive bacteria and gram-negative bacteria with stronger activity against gram-negative than gram-positive bacteria [17, 18]. *C. papaya* leaf extract inhibits the growth of *Salmonella typhi, Pseudomonas aeruginosa, Pseudomonas aureus, Shigella dysenteria,* and *Pseudomonas aeruginosa*. So the antibacterial activity adds to the advantage of carica papaya over Hemocoagulase which does not have such properties [19].

Due to its rich vascularity and salivary factors, any wound in the oral cavity tends to heal fast. Only in some cases does the healing of the wound require additional aid. The formation of a blood clot, its development into a structured matrix, and the deposition of bone are all necessary factors for the extraction site to recover normally. A dry socket will develop if these steps are violated [20,21].

Traditionally natural extracts have been used as home remedies for various ailments but the scientifically proven studies for these remedies have been not documented. More research on natural extracts and their impact on wound healing can bring these invaluable and age-old remedies to the limelight. This study proves that *Carica papaya* leaf extract showed better wound healing than Hemocoagulase in post-extraction sockets. More research on different extracts from papaya can give insight into new medicinal properties.

Limitations of the study

This study has proven the wound-healing effects of *Carica papaya* leaf extract and compared it with Hemocoagulase in the extraction socket of humans. Studies with larger sample size and histological analyses are required to analyze the efficiency and molecular mechanism of action of *Carica papaya* leaf extract.

## Conclusions

It can be concluded from the study that *Carica papaya* leaf extract showed better wound healing in post-extraction sockets compared to Hemocoagulase. This study presents the promising use of natural extracts such as* Carica papaya* in wound healing because they are easily accessible to patients, more economical, and have no adverse reactions. More studies that focus on natural extracts to promote wound healing are required in the future.
